# Continuous and synchronous calibration process of ovality and straightness for longitudinally submerged arc welding pipes with three rollers

**DOI:** 10.1371/journal.pone.0307293

**Published:** 2024-08-06

**Authors:** Xueying Huang, Yubin Zhang

**Affiliations:** 1 Tianjin University, Ministry Education, Key Lab Mech Theory & Equipment Design, Tianjin, People’s Republic of China; 2 School of Digital Technology and Engineering, Ningbo University of Finance and Economics, Ningbo City, People’s Republic of China; COMSATS University Islamabad, PAKISTAN

## Abstract

A new process of continuous and synchronous calibration process of ovality and straightness for LSAW (Longitudinally Submerged Arc Welding, LSAW) pipes with three rollers is proposed. Specifically, the process is introduced from three aspects: roller-shape, loading parameters and axial and circumferential deformation paths. The process is verified by numerical simulation and physical experiments. Further, the stress-strain in the Sections Ⅱ and Ⅳ is analyzed. The relationship between the process parameters and the residual ovality and residual straightness by experiments is discussed. The calibration scheme of LSAW pipes is put forward by using the control variable method. The results show that the shear stress is the principal stress direction in the Sections Ⅱ and Ⅳ. The residual ovality and residual straightness decrease with the increase of the radial reduction and times of reciprocating bending. The reciprocating bending process can eliminate the difference of the initial curvature, make the curvature of each section tend to be uniform. After calibration, the residual straightness is less than 0.2% and the residual ovality is less than 1%, demonstrating a good feasibility of this process.

## Introduction

With the development of the world’s oil and gas resources transferring to unconventionality, remote and harsh development environment and the development trend of oversized transmission have put forward higher requirements for pipeline construction. Longitudinally Submerged Arc Welding (LSAW) pipes are made from high-steel wide plates, especially for long-distance oil and gas pipeline transportation and for laying pipes in deep-sea or cold areas. The ovality and straightness of the formed welded pipe cannot meet the industrial standards due to the factors such as welding thermal stress, material properties and technical equipment [1–3]. Therefore, the formed welded pipe needs to be rounded and straightened (the process of rounding and straightening is referred to as the calibration process in this paper). According to the American Petroleum Institute’s industry standard ANSI/API Spec. 5L, the ovality and straightness of finished welding pipes have been strictly required. The ovality of the finished welding pipe does not exceed ±0.75% of the nominal outer diameter, and the straightness is not greater than 0.2% of the total length of the pipe [4].

At present, the ovality calibration process mainly includes the whole-diameter ovality calibration process [5,6], the over-bending ovality calibration process [7–9], and the roll-type ovality calibration process [10–12]. Common straightness calibration techniques include the pressure straightening [13–15] and the cross-roll straightening [16–19]. The existing ovality and straightness calibration processes are done separately. On the one hand, it leads to the growth of production process and the reduction of production efficiency, which is not easy to realize automatic and intelligent production; On the other hand, because the deformation of ovality calibration and straightness calibration affect each other, the flattening problem of LSAW pipes cannot be solved [20]. It is difficult to adjust both the ovality and straightness to optimal levels, which also seriously affects on-site welding and pipeline safety [21]. In view of the application trend of LSAW pipes and the existing problem of ovality calibration technology and straightness calibration technology, a new three-roller continuous and synchronous calibration process of ovality and straightness is proposed. The new process is based on the principle of axial and circumferential bidirectional reciprocating bending, combining the ovality calibration process with the straightness calibration process [21–24].

The presence of high external pressure may cause buckling in the circular section of subsea pipelines. It can further lead to buckling propagation and even overall collapse [25]. Therefore, the rounding process is very crucial. The stress-strain distribution during the ovality calibration section is also a key factor. The residual stress in the pipe has a negative impact on the fatigue strength, stress corrosion resistance, service life and so on. If the residual stress distribution of the circumferential section of a pipe is not uniform, the ovality of the pipe will increase with the gradual release of the residual stress. As one of the most important processes before a pipe is put into production, it is necessary to study the stress and strain generated by the ovality calibration section [26–28].

Relevant scholars have studied the mechanical properties such as stress-strain and residual stress deeply. Yuhua et al. investigated welding cracks in micro laser-welded NiTiNb shape memory alloy and Ti6Al4V alloy dissimilar metals joints, providing valuable insights into the challenges of welding dissimilar metals, which is relevant to understanding the stress and deformation behaviors in welded structures [29]. Chen et al. discussed the effects of post-weld heat treatment on the microstructure and mechanical properties of laser-welded NiTi/304SS joints with Ni filler. It provided a broader understanding of the microstructural changes and mechanical property improvements possible with post-weld treatments [30]. Deng et al. presented a prediction model for the ultimate forming dimensions in the ring rolling process, which was pertinent to understanding deformation processes similar to those investigated in the calibration of LSAW pipes [31]. Hua et al. explored the mechanism of void healing in cold-rolled M50 bearing steel under electroshocking treatment through a combined experimental and simulation study. It contributed to the understanding of stress management and defect mitigation in metal processing [32]. Liu et al. provided a foundation for understanding the modeling techniques on mathematical modeling and analysis of the tailor-rolled blank manufacturing process that can be applied to similar deformation and calibration processes [33]. Zhu et al. presented an improved method for measuring welding residual stress, which was crucial for ensuring the structural integrity and performance of welded components, directly relevant to residual stresses in LSAW pipes [34]. Hu et al. provided insights into the fatigue behavior of high-entropy alloys, which was relevant for understanding material durability in LSAW pipes [35]. Zhang et al. discussed interface behavior and nanoindentation characteristics in microbumps, offering comparative data on stress-strain behaviors [36]. Gao et al. explored elastic properties of auxetic structures, informing the deformation paths in LSAW pipes [37]. Wu et al. examined the impact of boron on structural stability in TLP joints, directly applicable to your calibration processes [38]. Additionally, Fang et al. provided valuable data on stress and strain behaviors during laser metal deposition, which could enhance the analysis of residual stresses [39]. Wang et al. and Liu et al. offered insights into mechanical performance and analytical methods [40,41]. Lastly, Long et al. presented findings on dynamic properties and predictive frameworks using machine learning, which could improve the analytical techniques in this research [42,43]. The above research has certain reference value for the understanding and study of the calibration process and mechanical properties of LSAW pipes in this paper.

The roller shapes, loading parameters, circumferential and axial deformation paths of the process are described in this paper. The distribution of stress-strain during the ovality calibration section is introduced by numerical simulation. The influencing factors of residual straightness and residual ovality are discussed. The feasibility of the process is verified by numerical simulation and physical experiments. The experimental platform is constructed and experiments are carried out. The calibration scheme for this process is proposed by using the control variable method.

### Process introduction

**Fig 1:**
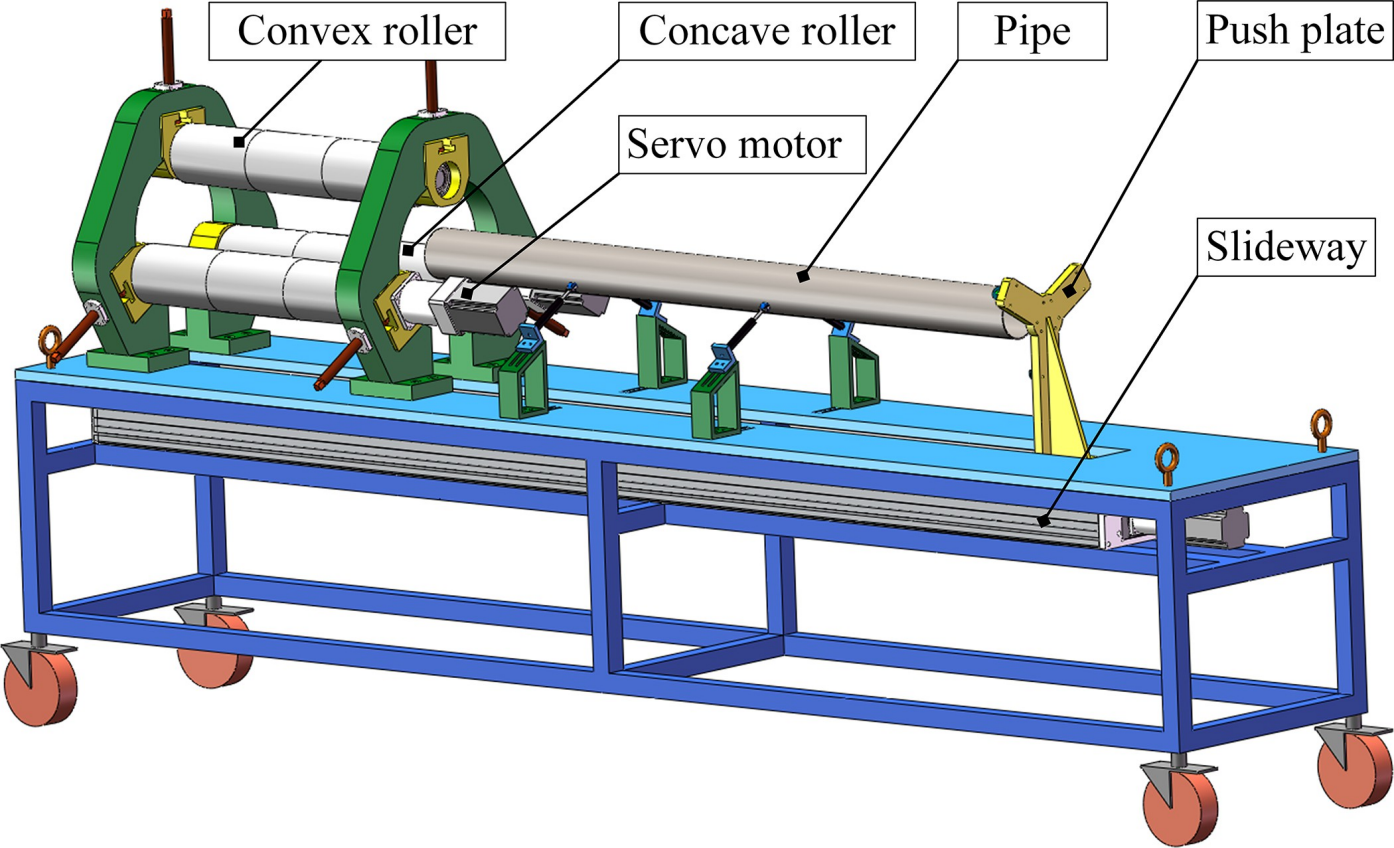
Schematic diagram of the calibration process.

The continuous and synchronous calibration process of ovality and straightness for LSAW pipes with three rollers is shown in Fig 1. The process’ main working parts are three parallel rollers, including a convex roller (upper roller) and two concave rollers (lower rollers). The whole process is described as follows:

Depending on the size of the pipe, adjust the position of the three rollers so that the axes of each roller are parallel to each other. Then, push each roller with the same radial reduction toward the central axis of the pipe.

Two concave rollers rotate simultaneously driven by servo motors. This causes the pipe and convex roller to be driven to rotate under the action of friction. At the same time, the servo motor drives the push plate to make the pipe move along the slideway, thus enabling the calibration process.

Finally, the pipe is pushed out continuously until it is separated from three rollers. Each roller stops rotating this moment. The push plate moves automatically towards the opposite direction until it returns to its initial position, to complete the whole semi-automatic process.

### Roller-shape

**Fig 2:**
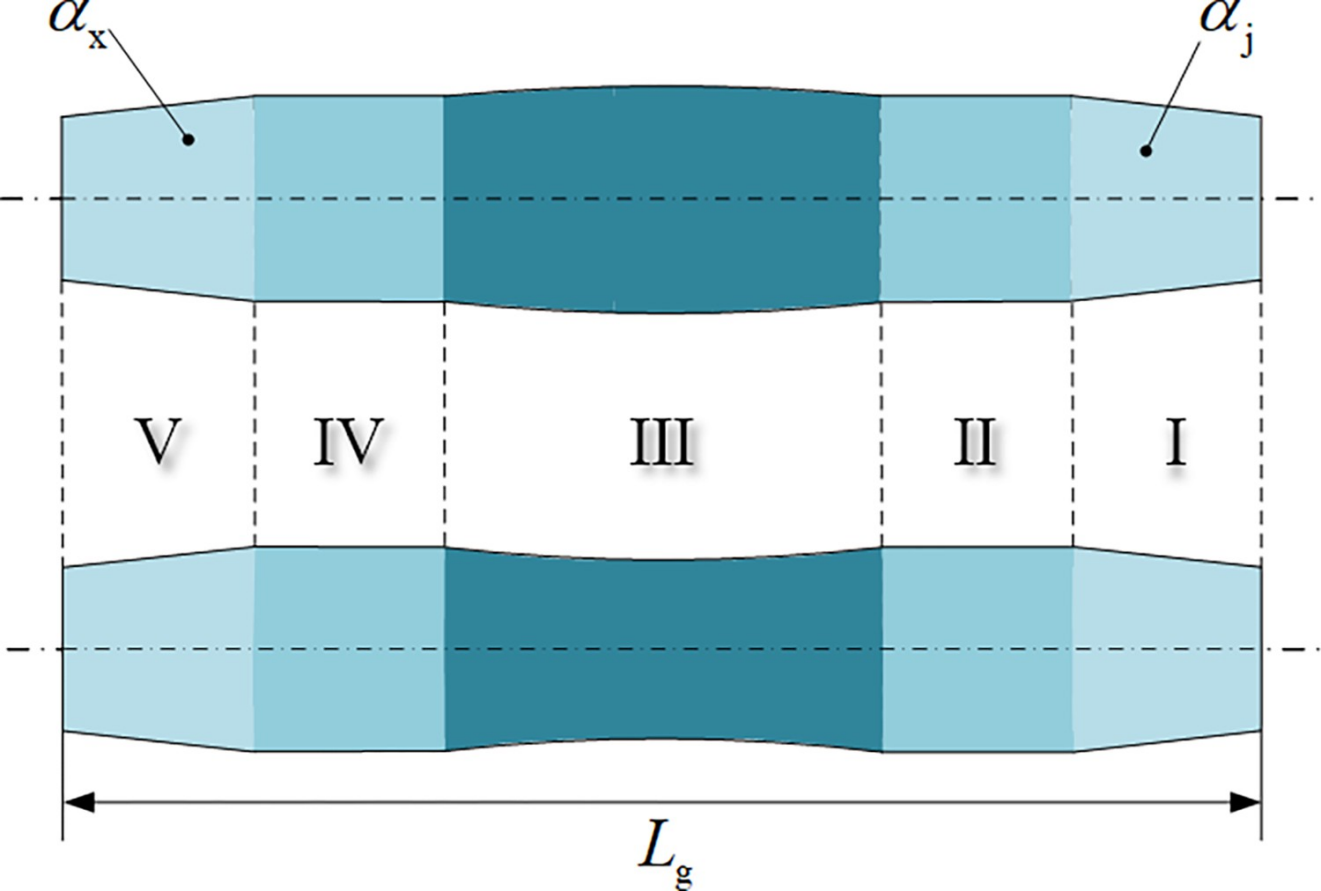
Schematic diagram of roller-shape. Section Ⅰ: Loading section; Section Ⅱ: Ovality calibration section; Section Ⅲ: Ovality and straightness calibration section; Section Ⅳ: Ovality complement calibration section; Section Ⅴ: Unloading section.

The schematic diagram of roller-shape is shown in Fig 2. Each roller is divided into five sections: loading section, ovality calibration section, ovality and straightness calibration section, ovality complement calibration section and unloading section.

As shown in Fig 2, both ends of the roller are loading section (Section Ⅰ) and unloading section (Section Ⅴ), both of which are truncated cone shape. The design enables the pipe to enter smoothly between the three rollers and ensures that the pipe can unload smoothly, so as to unify the curvature.

The taper of loading section and unloading section are respectively [23]

$$\alpha_{j}=\frac{3(2H_{\max}+2a-D_{p})}{L_{g}}$$ (1)

$$\alpha_{x}=\frac{6H_{\max}}{L_{g}}$$ (2)

Where *α*_j_ is the taper of loading section, *α*_x_ is the taper of unloading section, *H*_max_ is the maximum radial reduction allowed for pipe calibrating, *a* is the major axis diameter of the pipe, *D*_p_ is the nominal outside diameter of the pipe, *L*_g_ is the length of roller.

Section Ⅱ and Section Ⅳ are ovality calibration sections, which are cylindrical in shape. The design can make the pipe undergo multiple reciprocating bending along the circumferential direction, resulting in elastic-plastic deformation. Thus, ovality calibration and complementary ovality calibration are realized.

Section Ⅲ is ovality and straightness calibration section. The upper roller part is convex and the lower roller part is concave. The design ensures that the pipe can undergo multiple reciprocating bending along the circumferential and axial directions. Thereby, ovality and straightness calibration are realized.

### Loading parameter

**Fig 3:**
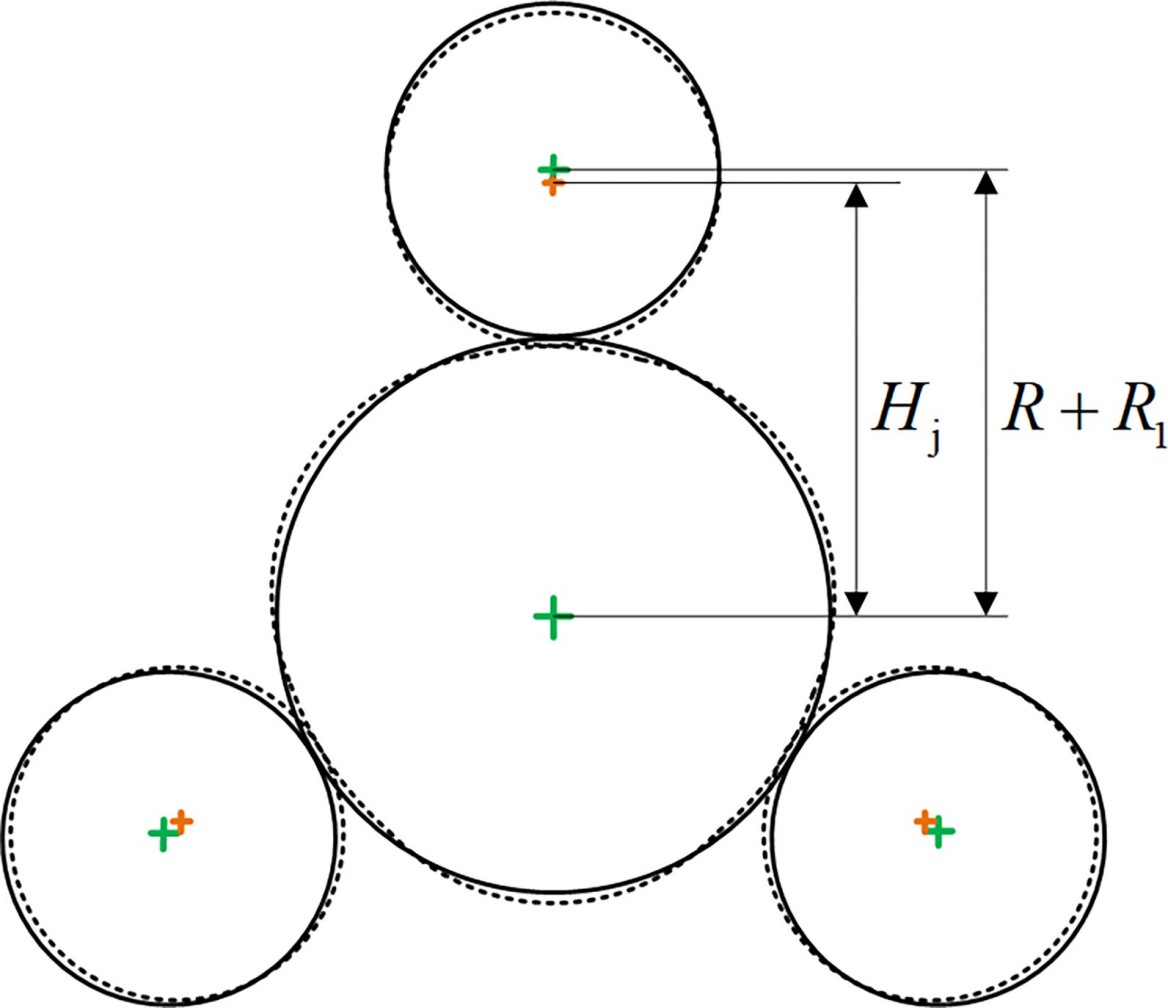
Diagram of loading parameters.


Fig 3 is a schematic diagram of the loading parameter. The three rollers load the same radial reduction toward the pipe center, and the stroke of each roller is recorded as *H*.

$$H=R_{1}+R-H_{j}$$ (3)

where *H* is the radial reduction, *R*_1_ is the roller radius, *R* is the pipe radius, *H*_j_ is the distance from the pipe’s center to the center of roller after loading.

### Deformation path of the axial reciprocating bending process

**Fig 4:**
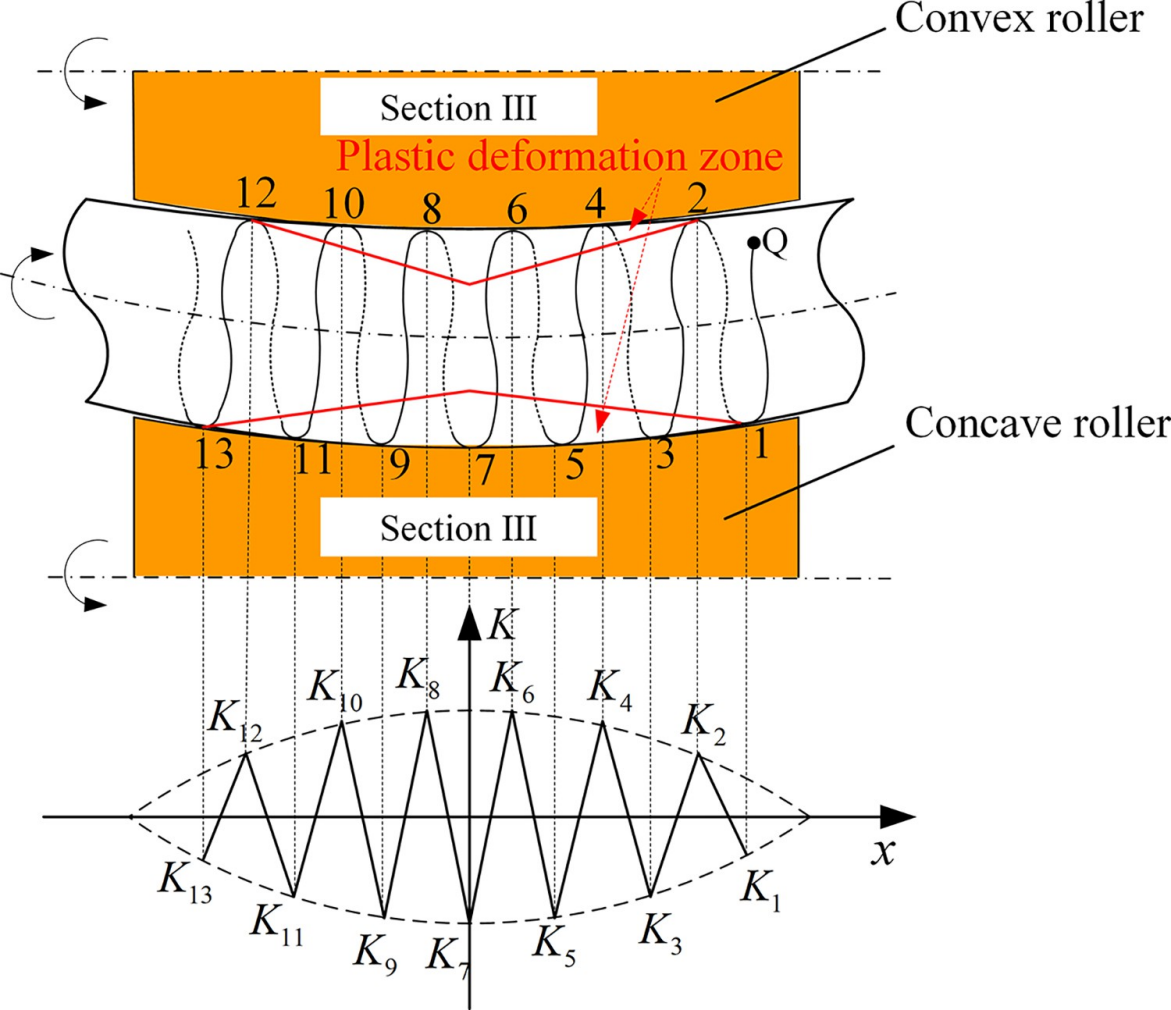
Deformation path of particle along axial direction.

Since the pipe rotates and advances axially at the same time, any particle of the pipe alternately undergoes multiple positive-reverse bending process. The straightening process is an axial reciprocating bending process. The deformation path of the pipe particle along the axial direction is shown in Fig 4. The restraint and compression of the pipe by three rollers make it produce elastoplastic deformation within a certain range. To describe the reciprocating bending deformation process, any particle of the pipe is tracked. The trajectory of the particle **Q** is a spiral. Its center of rotation is the pipe axis. The deformation outside the plastic deformation zone is elastic deformation and has no influence on straightening.

As can be seen from Fig 4, particle **Q** reciprocates from position 1 to position 13. A total of 13 plastic bends is experienced throughout the straightening process. In the plastic deformation zone, the cross-sections at positions 2, 4, 6, 8, 10 and 12 are bent in a positive direction, and the cross-sections at positions 1, 3, 5, 7, 9, 11 and 13 are bent in a reverse direction. Since the Section Ⅲ is a curve of variable curvature, the absolute value of curvature increases first and then decreases. That is, $\left|K_{1}|\to |K_{2}|\to |K_{3}|\to |K_{4}|\to |K_{5}|\to |K_{6}|\to |K_{7}\right|$ increases gradually, $\left|K_{7}|\to |K_{8}|\to |K_{9}|\to |K_{10}|\to |K_{11}|\to |K_{12}|\to |K_{13}\right|$ decreases gradually, and *K*_1_ = *K*_13_, *K*_2_ = *K*_12_, *K*_3_ = *K*_11_,……, *K*_6_ = *K*_8_.

### Deformation path of the circumferential reciprocating bending process

**Fig 5:**
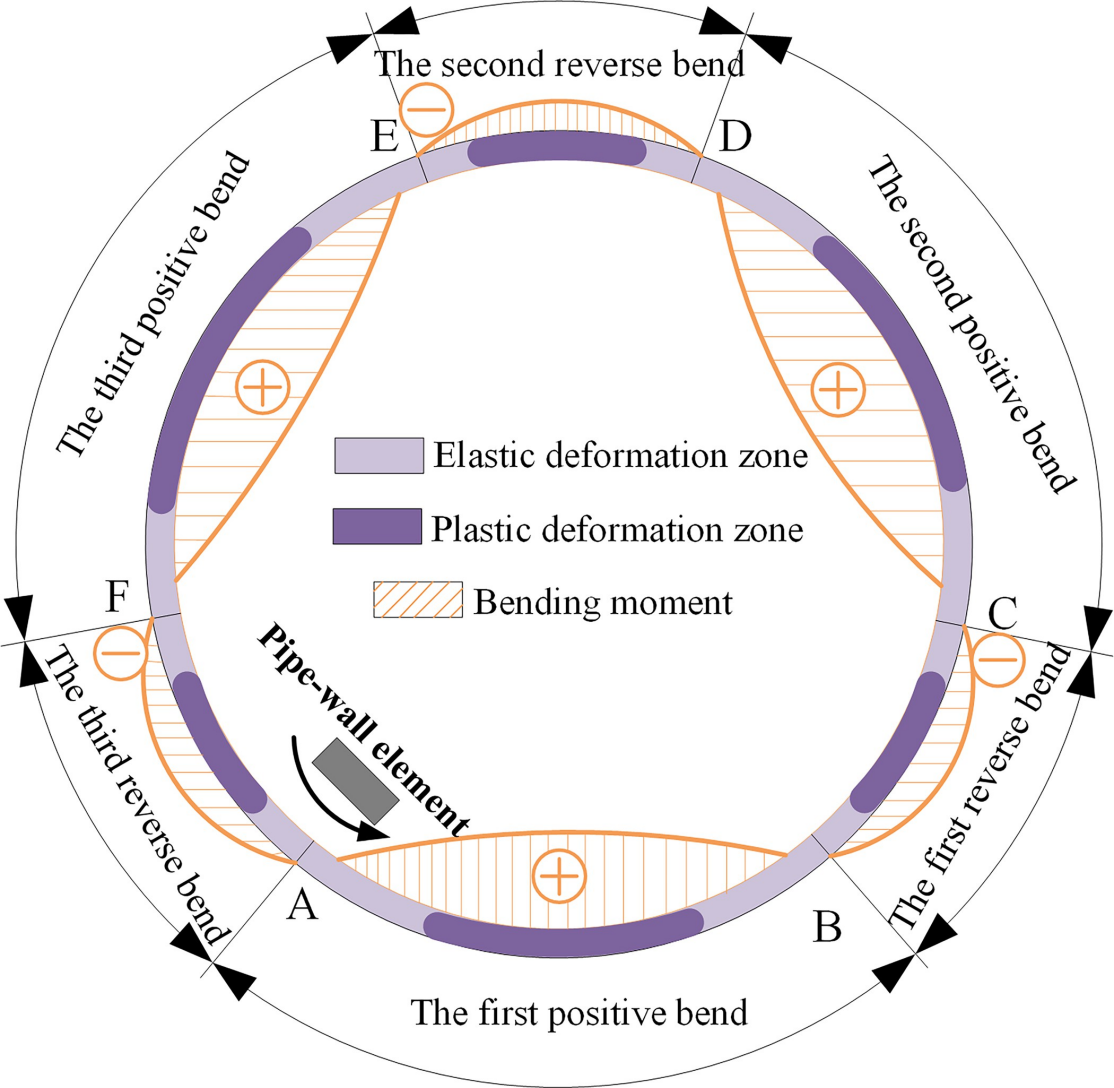
Deformation path of particle along circumferential direction.

Under the action of friction, the pipe does a rotational motion. Its circumferential cross-section alternately undergoes multiple positive bending process and reverse bending process. The pipe rounding process is a circumferential reciprocating bending process. Fig 5 shows the deformation trajectory of the pipe particle along the circumferential direction. The pipe is constrained and compressed by three rollers, resulting in elastoplastic deformation of the pipe in both positive and reverse bending zones. To describe the circumferential reciprocating bending deformation process, a section of the pipe-wall element of the pipe is selected.

With one rotation starting from position A, the continuous deformation process of the pipe-wall element is as follows:

1. The first positive bend: elastoplastic loading and unloading from position A to position B.
2. The first reverse bend: elastoplastic loading and unloading from position B to position C.
3. The second positive bend: elastoplastic loading and unloading from position C to position D.
4. The second reverse bend: elastoplastic loading and unloading from position D to position E.
5. The third positive bend: elastoplastic loading and unloading from position E to position F.
6. The third reverse bend: elastoplastic loading and unloading from position F to position A.

### Numerical simulation and experimental design

**Fig 6:**
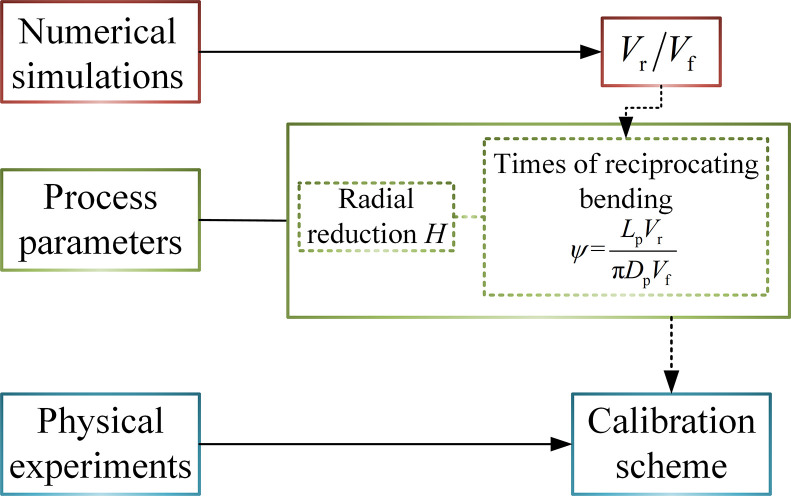
Flow chart for formulating the calibration scheme.

The flow chart for formulating the calibration scheme by numerical simulations and physical experiments is shown in Fig 6. As mentioned above, radial reduction and times of reciprocating bending are the main process parameters. To determine the times of reciprocating bending, it is necessary to solve the ratio of roller rotation speed (*V*_r_) to pipe forward speed (*V*_f_). (Fig 6) The ratio is discussed based on numerical simulation analyses. Then, the times of reciprocating bending is obtained. Based on the control variable method, the influence of process parameters on the residual ovality and residual straightness is obtained through experiments. Combining the above analysis results, a solution is proposed, that is, the calibration scheme of the process.

The number of reciprocating bending is calculated from the beginning of contact with three rollers to completely leaving three rollers. The number of reciprocating bending cycles was determined through the roller rotation speed (*V*_r_) and the pipe forward speed (*V*_f_). The equation to be solved is as follows,

$$\psi =\frac{L_{p}V_{r}}{\pi D_{p}V_{f}}$$ (4)

Where, *ψ* is the number of reciprocating bending; *V*_r_ is the roller rotation speed (mm/s); *V*_f_ is the pipe forward speed (mm/s); *L*_p_ is the pipe length (mm); *D*_p_ is the pipe outer diameter (mm).

### Numerical simulation

**Fig 7:**
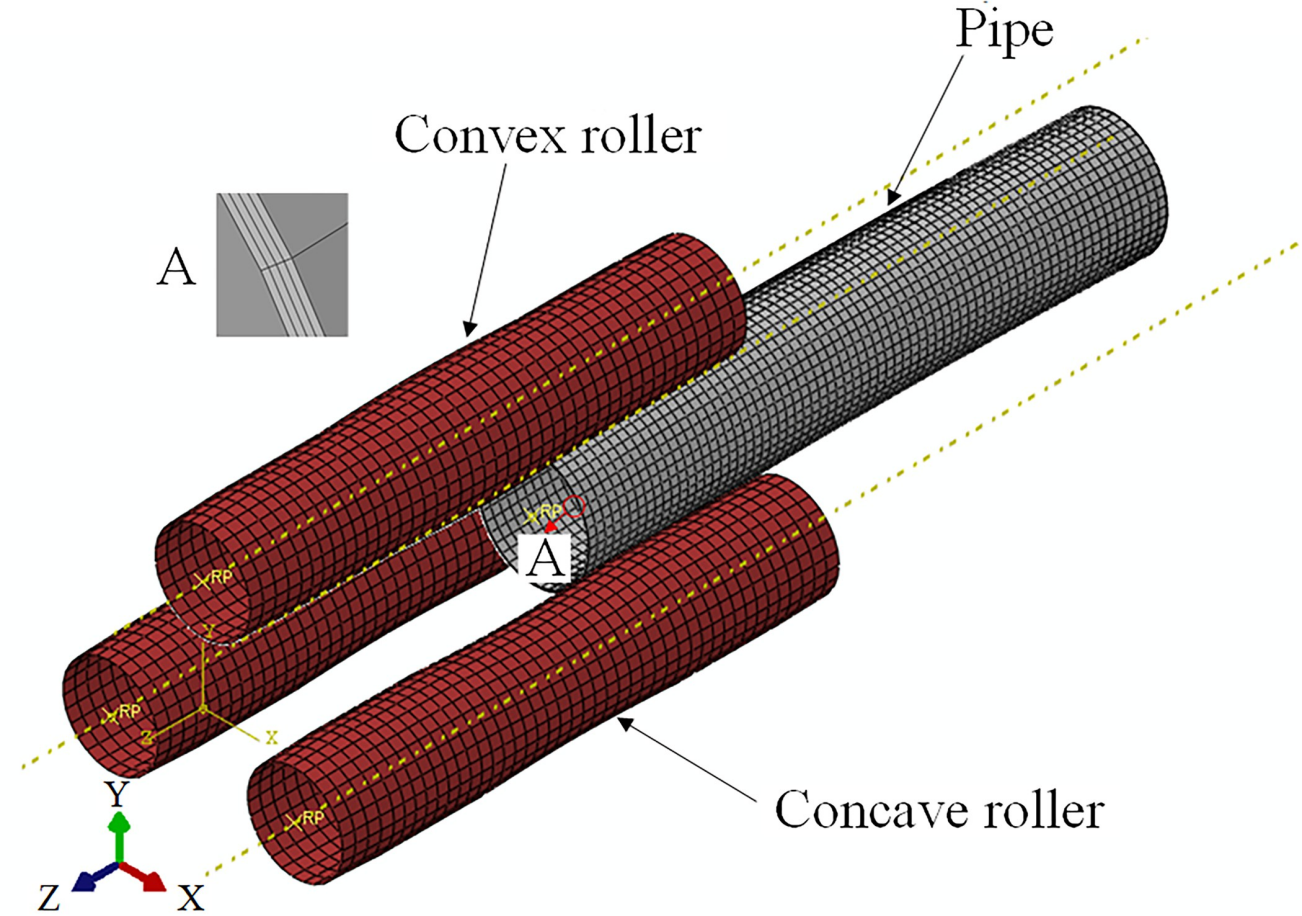
Finite element model.

Using the software ABAQUS, a finite element model of the LSAW pipe calibration process is established, as shown in Fig 7. Regardless of the pipe welds on the continuous calibration process, the mechanical properties and geometric dimensions of pipes are shown in Table 1 and the geometric dimensions of rollers is shown in Table 2. The pipe is set as a deformable body. The pipe-wall is divided into 4 layers along the thickness. The pipe is discretized by 8-node linear hexagonal nonconforming mode elements. Select the continuum distributing coupling method to couple the center point of the pipe and the boundary. Six degrees of freedom for the constrained area is selected. The three rollers are set as discrete rigid bodies. The contact between the pipe and each roller is set to pure master-slave contact and motion contact conditions. The coefficient of friction is 0.2.

**Table 1:** Mechanical properties and geometric dimensions of pipes.

Material	Elastic modulus *E* (GPa)	Yield stress *σ*_s_ (MPa)	Plastic modulus *D* (MPa)	Outer diameter *D*_p_ (mm)	Length *L*_p_ (mm)	Thickness *t* (mm)	Initial ovality	Initial straightness
304	234	294	2842	140/160	1000	2/1.5	5%	10‰

**Table 2:** Geometric dimension of rollers.

Outer diameter *D*_g_ (mm)	Length *L*_g_ (mm)	Proportion of rollers	Taper of Section Ⅰ (rad)	Taper of Section Ⅴ (rad)	*K*_*n*_ (mm^-1^)	Roller shape curve of Section Ⅲ
120	600	1:2:4:2:1	0.033	0.025	0.001	0.16*x*^2^−0.0004*y*^2^−1 = 0

### Experimental design

**Fig 8:**
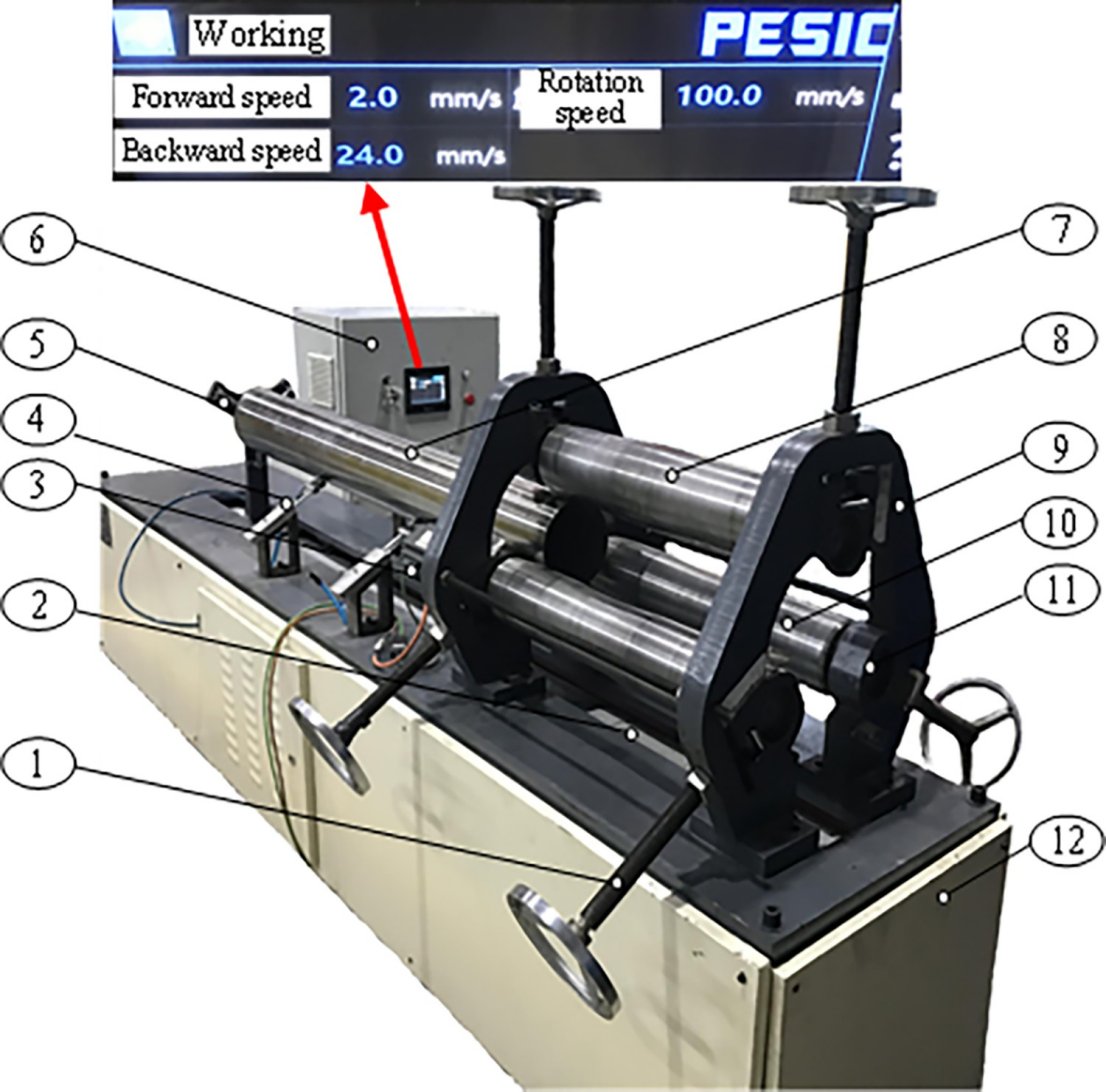
Experimental device for pipe calibration. 1. Screw 2. Lead screw drive 3. Servo motor 4. Support assembly 5. Push plate 6. Control cabinet 7. Pipe 8. Upper roller 9. Frame 10. Lower roller 11. Slider 12. Pedestal.

The experimental device for pipe calibration is shown in Fig 8. The device can realize both continuous rounding and continuous straightening of LSAW pipes.

The roller is connected with the slider fixed on the frame via bearing (Fig 8). The slider can slide vertically along the frame surface via a screw to adjust the radial reduction of three rollers. The support assembly keeps the balance of the pipe during the experiment. The servo motor drives two lower rollers to rotate synchronously, which drives the pipe and the upper roller to start turning. At the same time, the push plate drives the pipe to move along the slideway. So far, the calibration process of simultaneous rotation and movement of the pipe is realized. The pipes and rollers selected for the experiment are the same as the pipes and rollers selected for the simulation.

## Results and discussion

### Simulation results

#### Ovality calibration section (Sections Ⅱ and Ⅳ)

**Fig 9:**
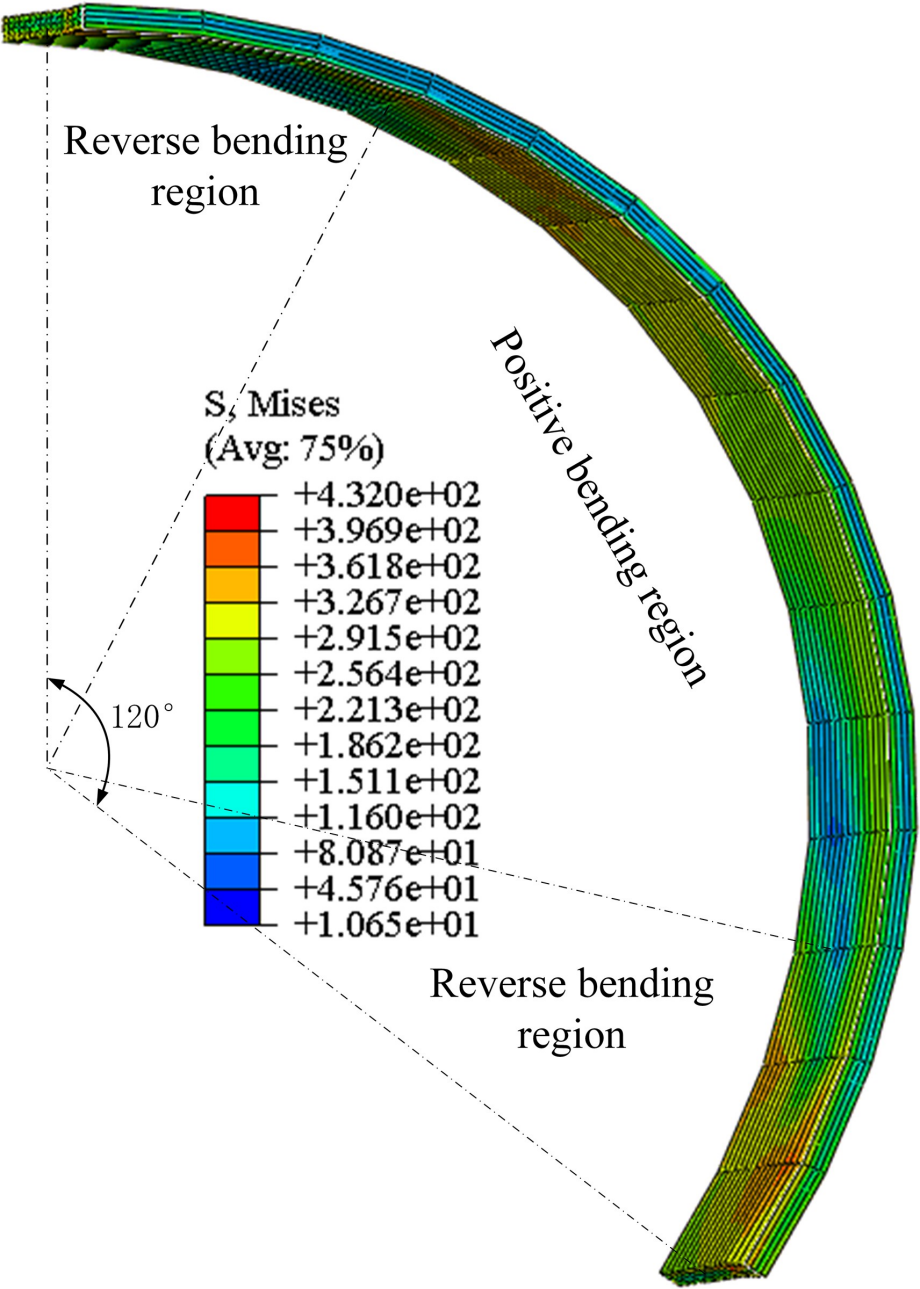
Distribution of equivalent stress along the one-third pipe.

The equivalent stress distribution along the one-third pipe is shown in Fig 9. According to the symmetry of the circle, there are three positive bending regions and three reverse bending regions.

**Fig 10:**
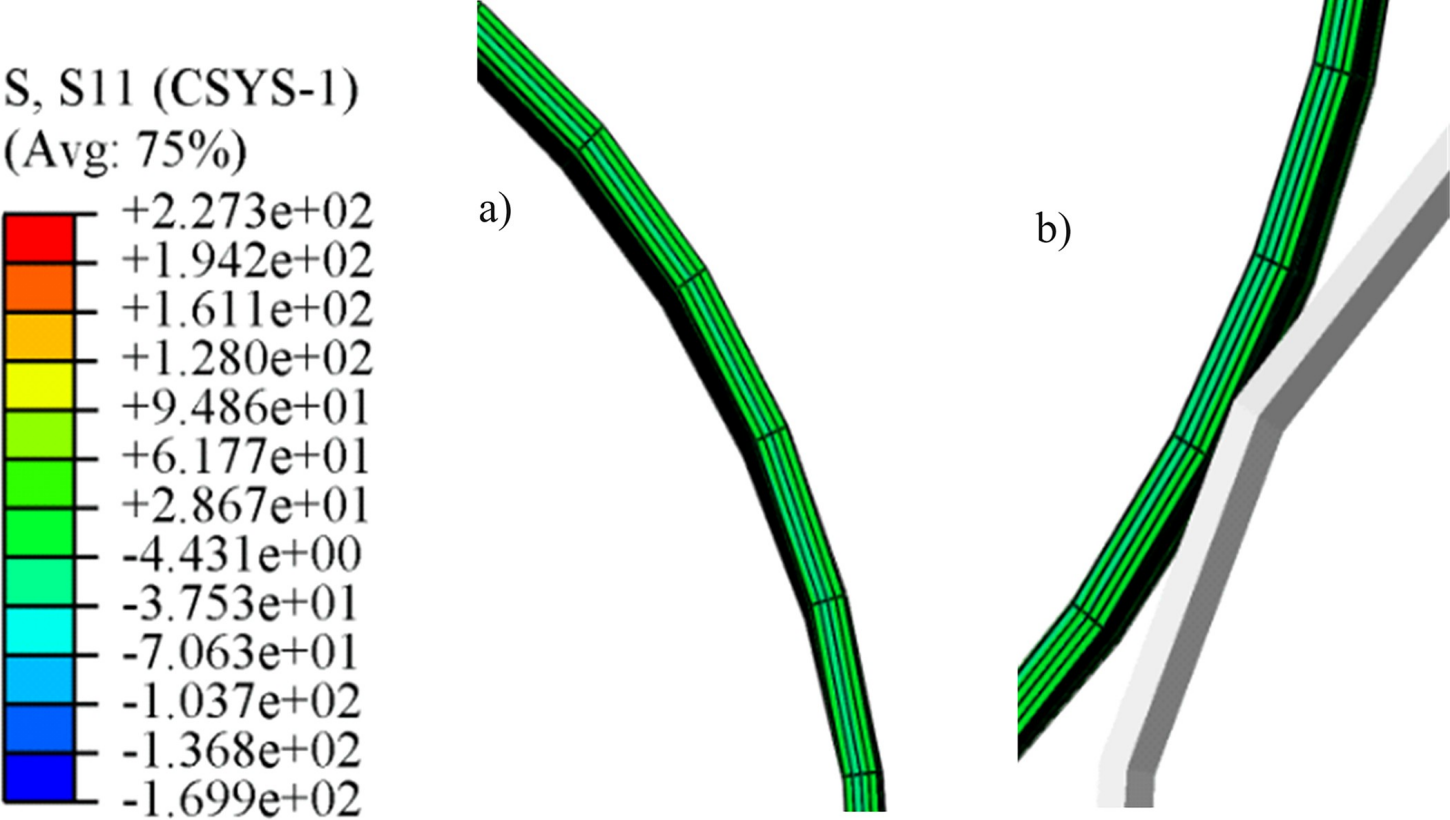
Distribution of radial stress along the thickness direction. a) Positive bending region; b) Reserve bending region.

The distribution of radial stress along the thickness direction is shown in Fig 10. The radial stress of the pipe is small and does not reach the yield stress of the material. So, it does not produce elastoplastic deformation. Therefore, it can be assumed that the geometric dimensions along the thickness of the pipe do not change during the ovality calibration section.

**Fig 11:**
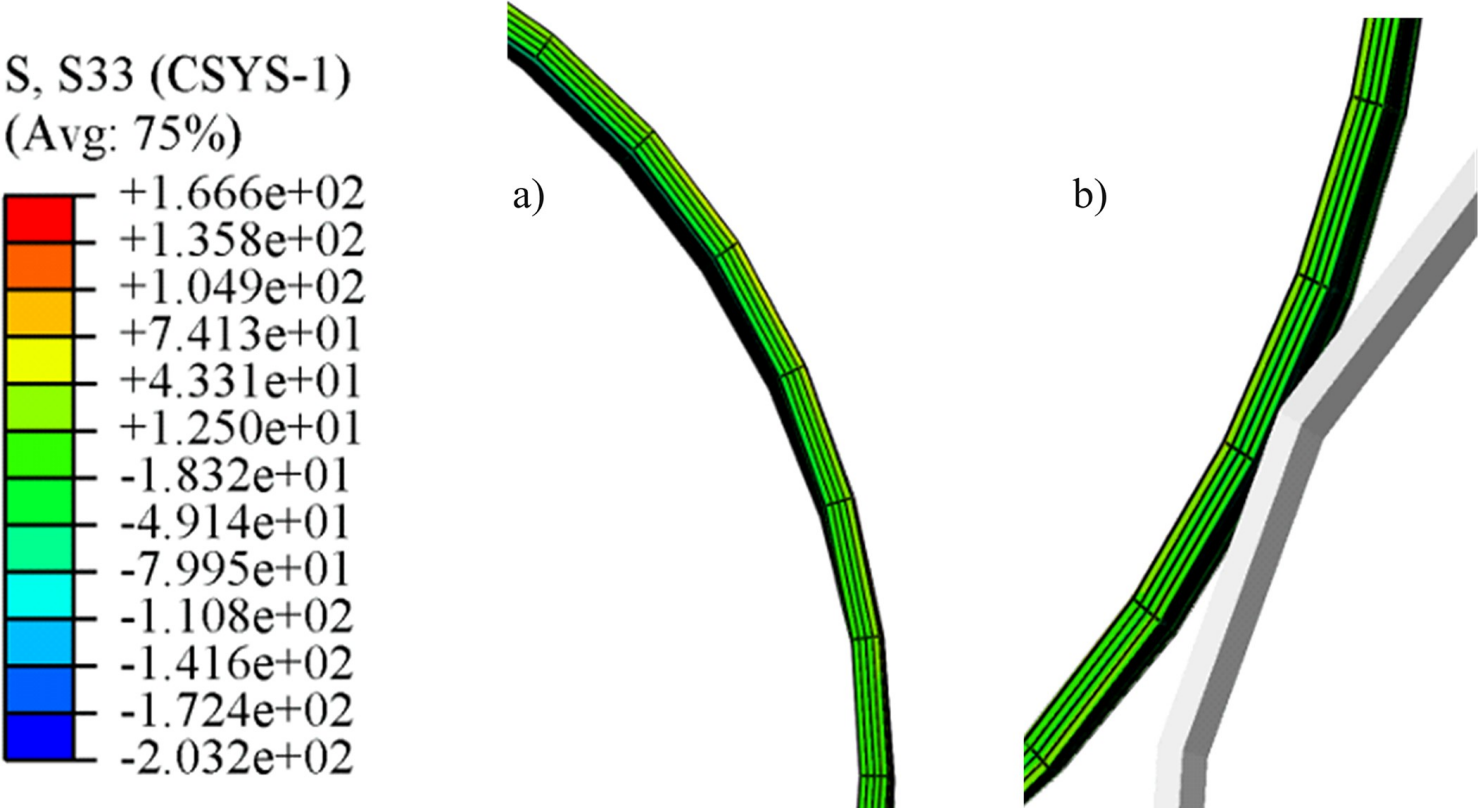
Distribution of axial stress along the thickness direction. a) Positive bending region; b) Reserve bending region.

The distribution of axial stress along the thickness direction is shown in Fig 11. The deformation behavior of the inner and outer material fibers of the pipe is inhibited. The axial stress of the pipe is very small and will not cause plastic deformation. During the ovality calibration section, the geometry does not change along the length of the pipe.

**Fig 12:**
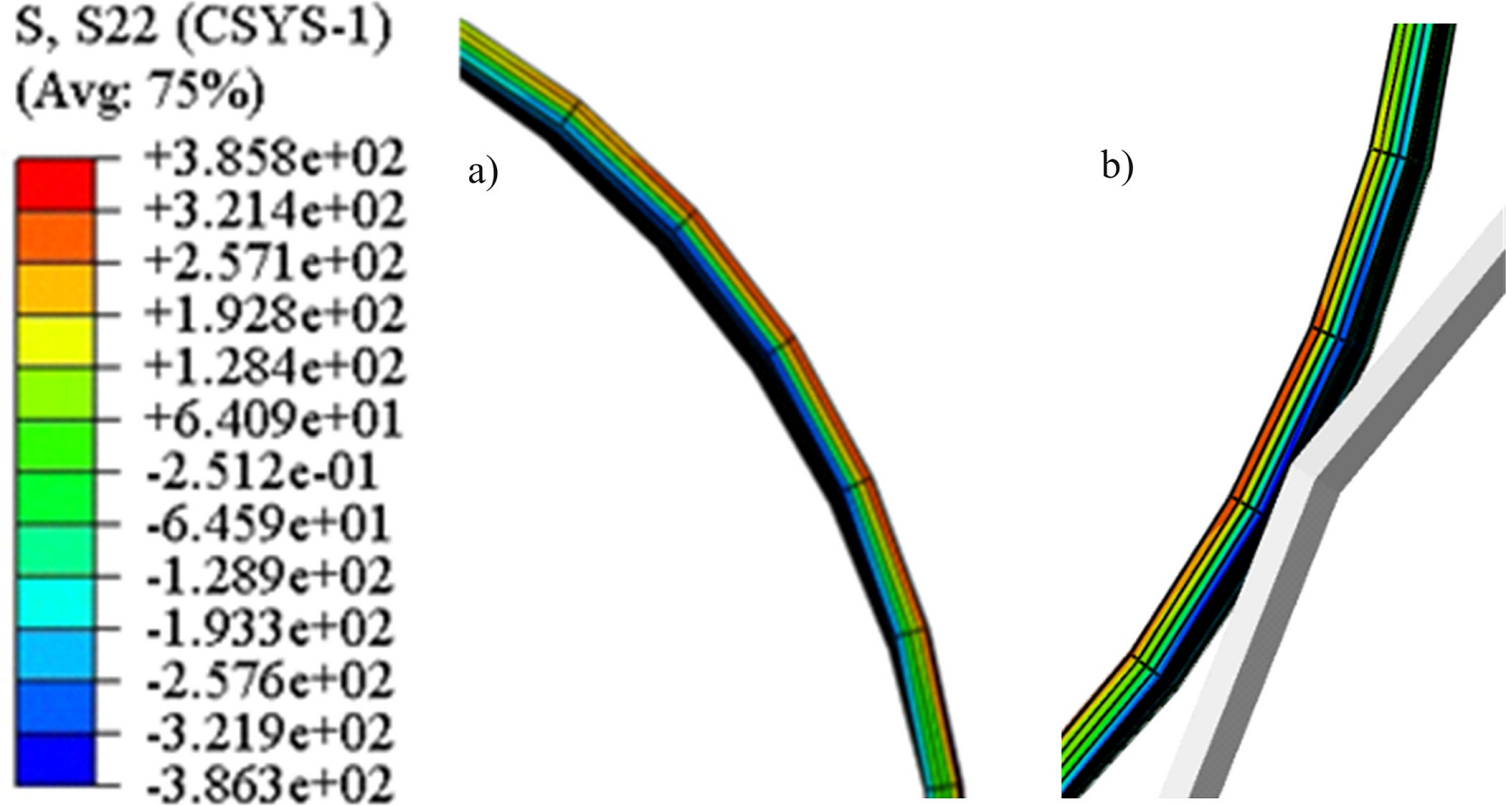
Distribution of shear stress along the thickness direction. a) Positive bending region; b) Reserve bending region.

The distribution of shear stress along the thickness direction is shown in Fig 12. In the positive bending region, the maximum stress value of the outer fiber is 321.4MPa, and the minimum stress value of the inner fiber is -321.9MPa. In the reverse bending region, the minimum stress value of the outer fiber is -386.3MPa, and the maximum stress value of the inner fiber is 385.8MPa. The shear stress causes the inner and outer layers of the pipe to enter the yield stage first, resulting in plastic deformation. Therefore, the direction in which the shear stress is located is the main stress direction.

**Fig 13:**
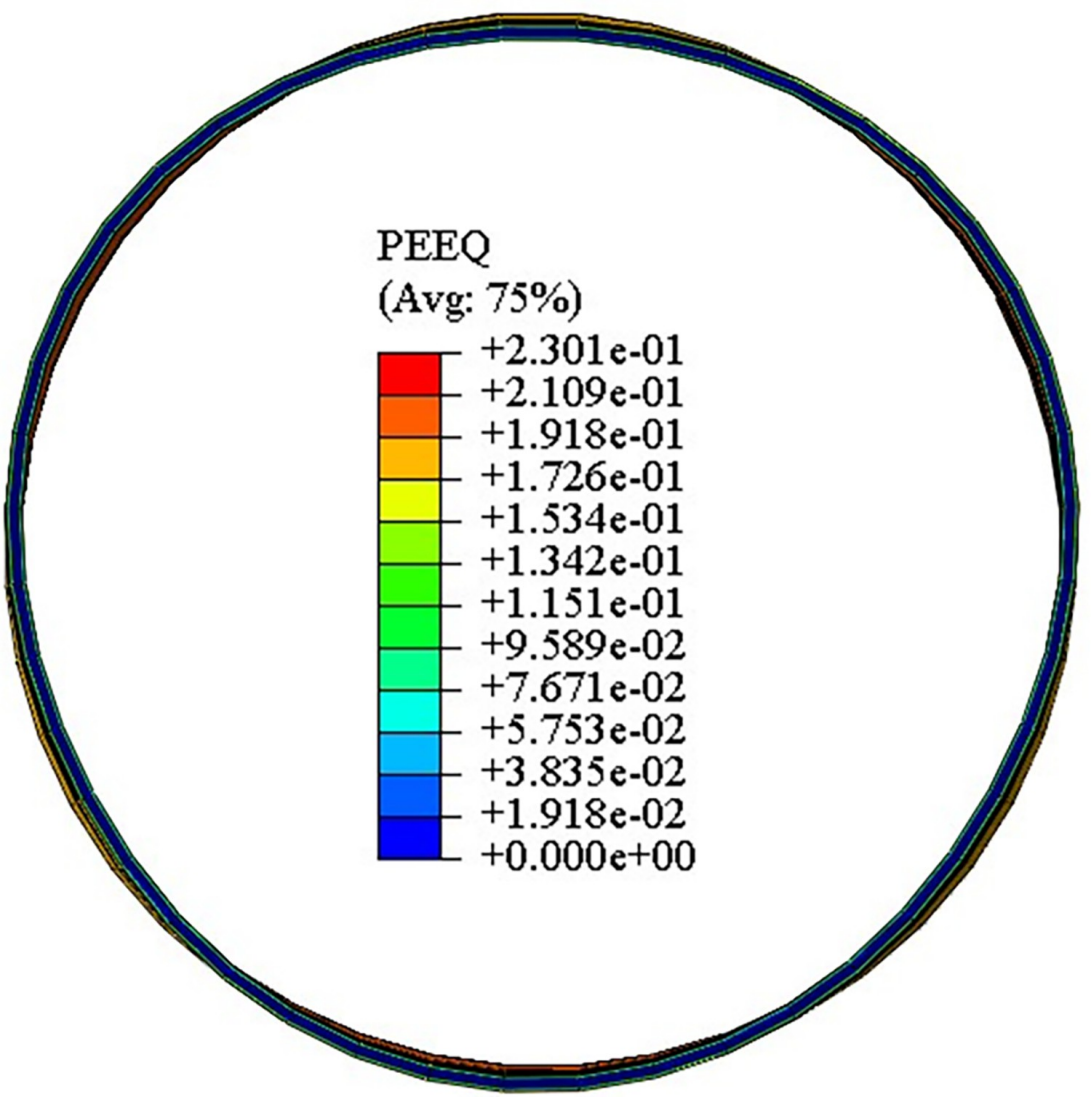
Distribution of equivalent strain in the ovality calibration section.

The distribution of equivalent strain in the ovality calibration section is shown in Fig 13. The deformation behavior of the pipe belongs to the small deformation. Its equivalent strain value is also small. The maximum equivalent strain value is 0.23 in the main deformation region and 0 in the geometrically neutral layer.

**Fig 14:**
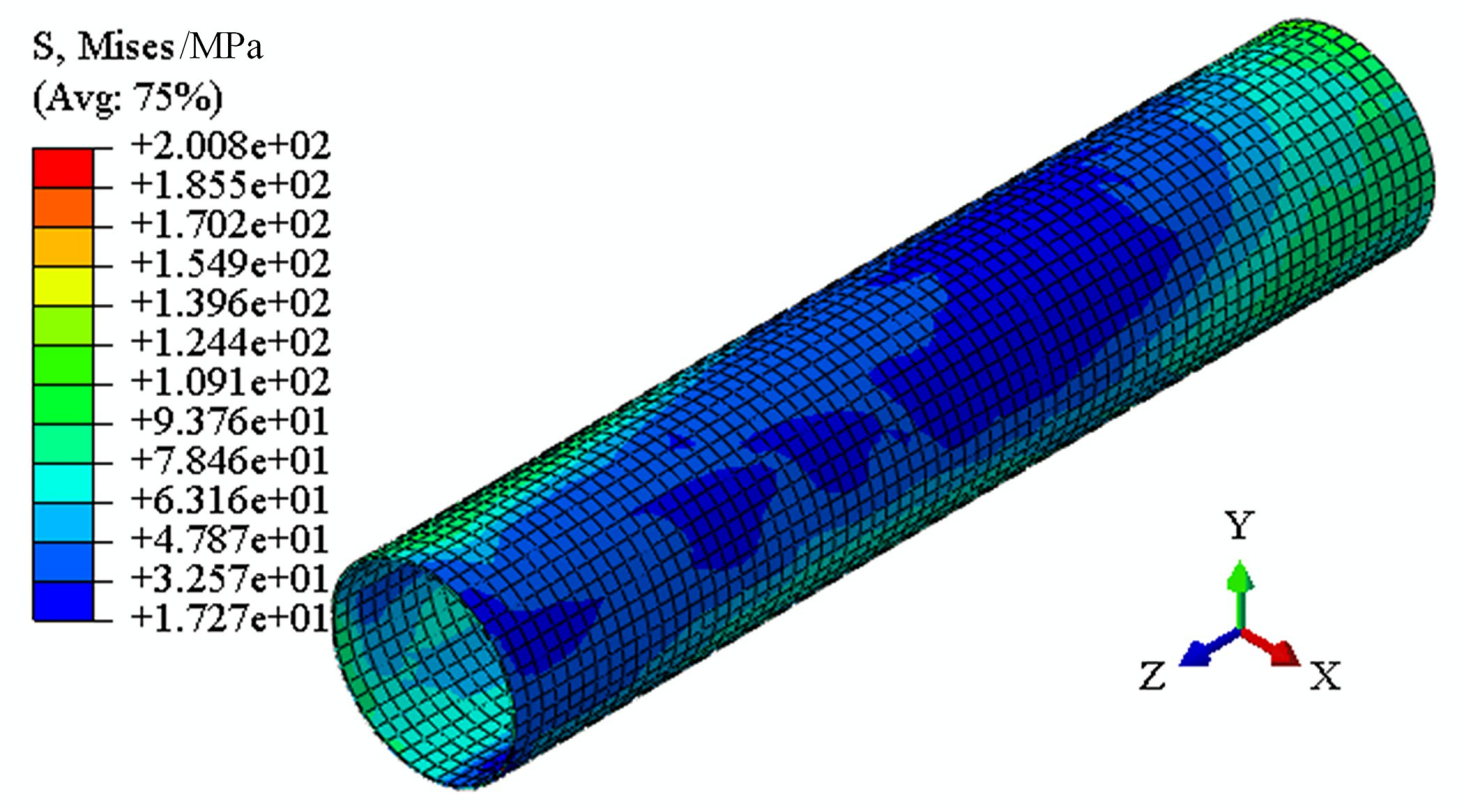
Distribution of residual stress of 304 pipe after calibration.

The equivalent stress distribution of 304 pipe after calibration is shown in Fig 14. The distribution of equivalent stress tends to be uniform and the residual stress is small.

#### Determination of key parameters

**Fig 15:**
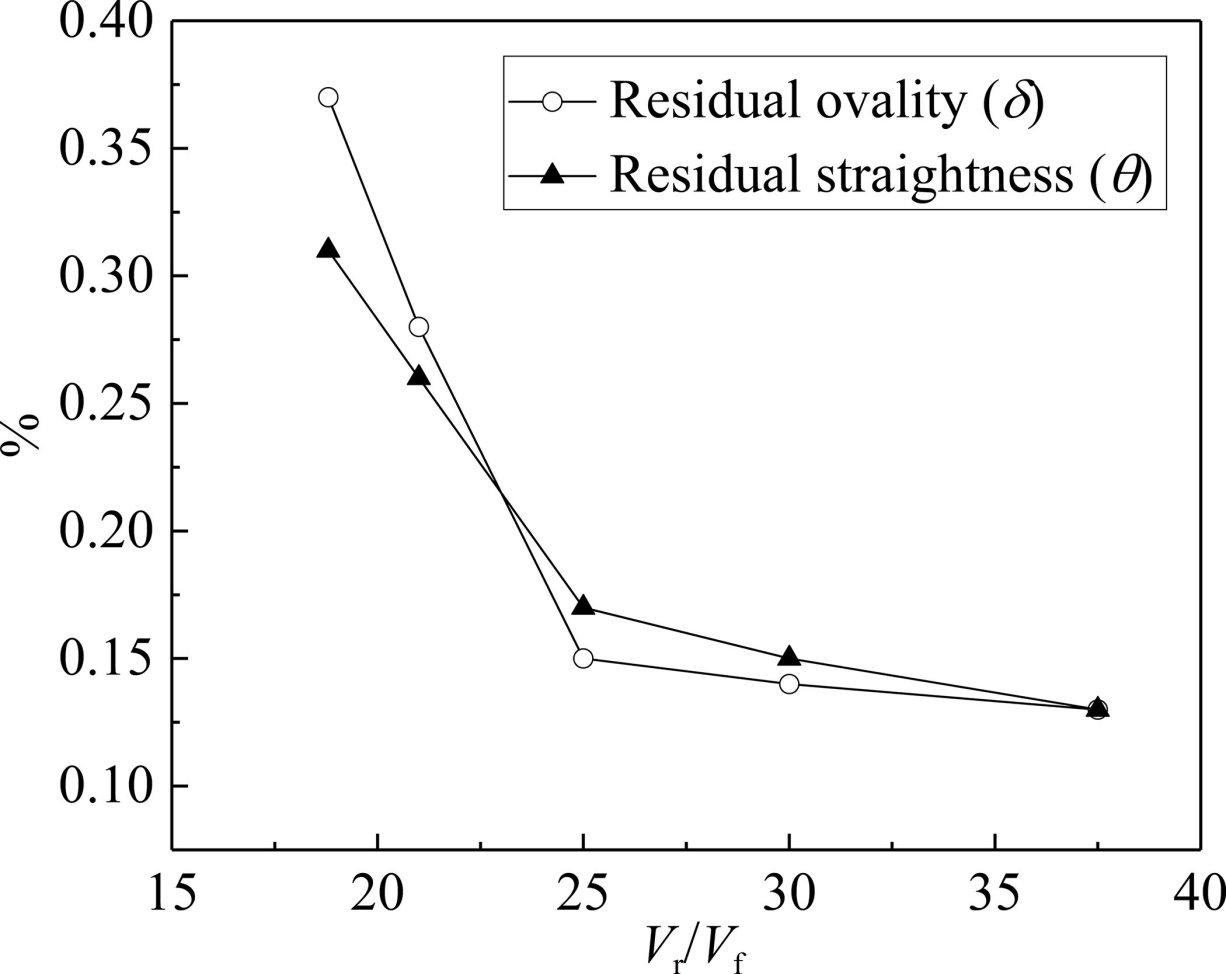
Effect of the ratio of *V*_r_ to *V*_f_ on residual ovality and residual straightness.

As can be seen from reference [12], the larger the diameter-thickness ratio of the pipe, the less likely it is to achieve calibration. To reduce the simulation times, the pipe with large diameter-thickness ratio is selected for analysis. In this paper, the pipe with a diameter of 160 mm and a thickness of 1.5 mm is selected as the research object. The ratio of roller rotation speed (*V*_r_) to pipe forward speed (*V*_f_) is determined by numerical simulation. The effect of the ratio of *V*_r_ to *V*_f_ on residual ovality and residual straightness is shown in Fig 15.

The residual ovality and residual straightness of the pipe gradually decrease with the increase of the ratio of *V*_r_ to *V*_f_. When *V*_r_/*V*_f_≥25, the residual ovality is within 1% and the residual straightness is within 2‰. The results meet the standard requirements [4]. As a result, the feasibility of the process is initially verified. It should be noted that the ratio should not be less than 25 in the experiment.

#### Experimental results

**Fig 16:**
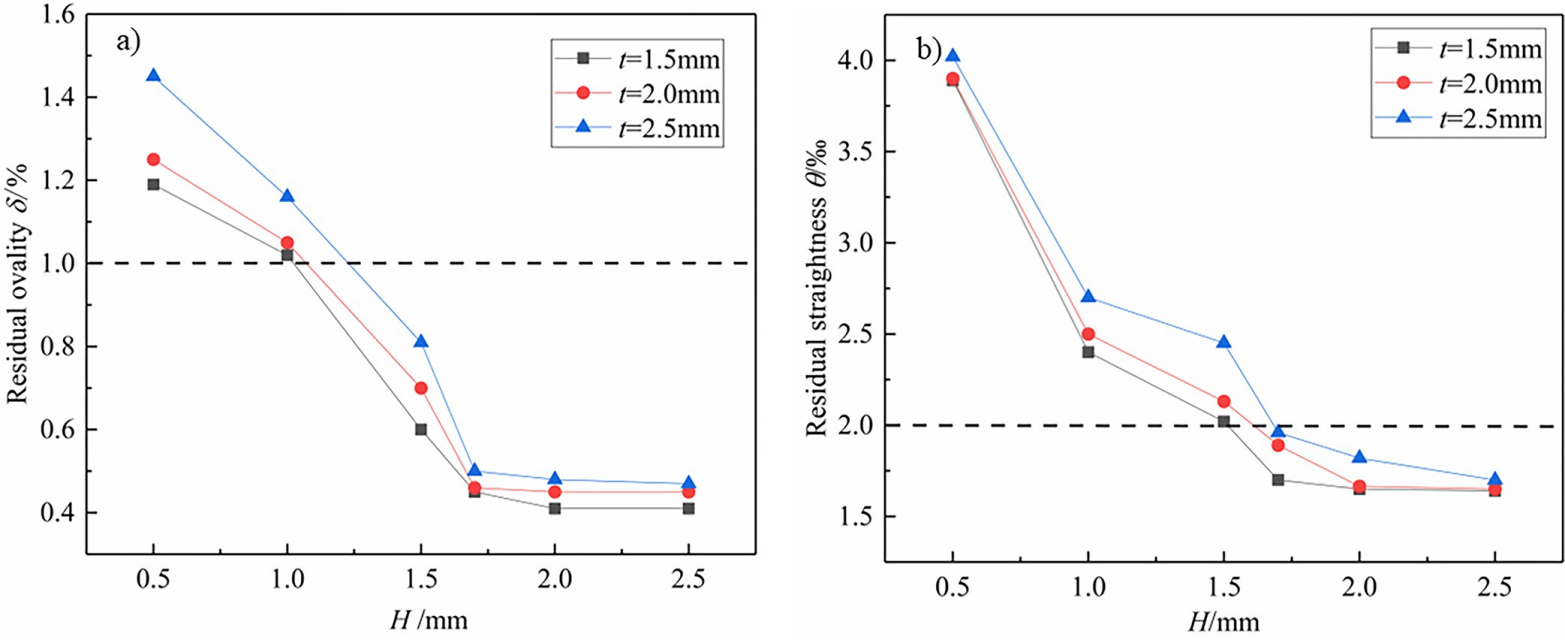
Effect of radial reduction on residual ovality and residual straightness of pipes. a) Residual ovality; b) Residual straightness; the times of reciprocating bending *ψ* is 50.

According to the above simulation results, the ratio of *V*_r_ to *V*_f_ has been determined. When the the ratio of *V*_r_ to *V*_f_ is 25, the radial reduction (*H*) of pipes is discussed. The pipes with a diameter of 160 mm with a thickness of 1.5 mm, 2.0 mm and 2.5mm are selected as the research object. The effect of radial reduction on residual ovality and residual straightness of pipes is shown in Fig 16. It can be observed that the residual ovality and residual straightness gradually decrease with the increase of radial reduction. Fig 16A shows that the residual ovality of the pipe is within 1% when *H* > 1.5 mm. Fig 16B shows that the residual straightness of the pipe is within 0.2% when *H* > 1.7 mm. In conclusion, the residual ovality and residual straightness of the above pipes meet the industry standards [4].

**Fig 17:**
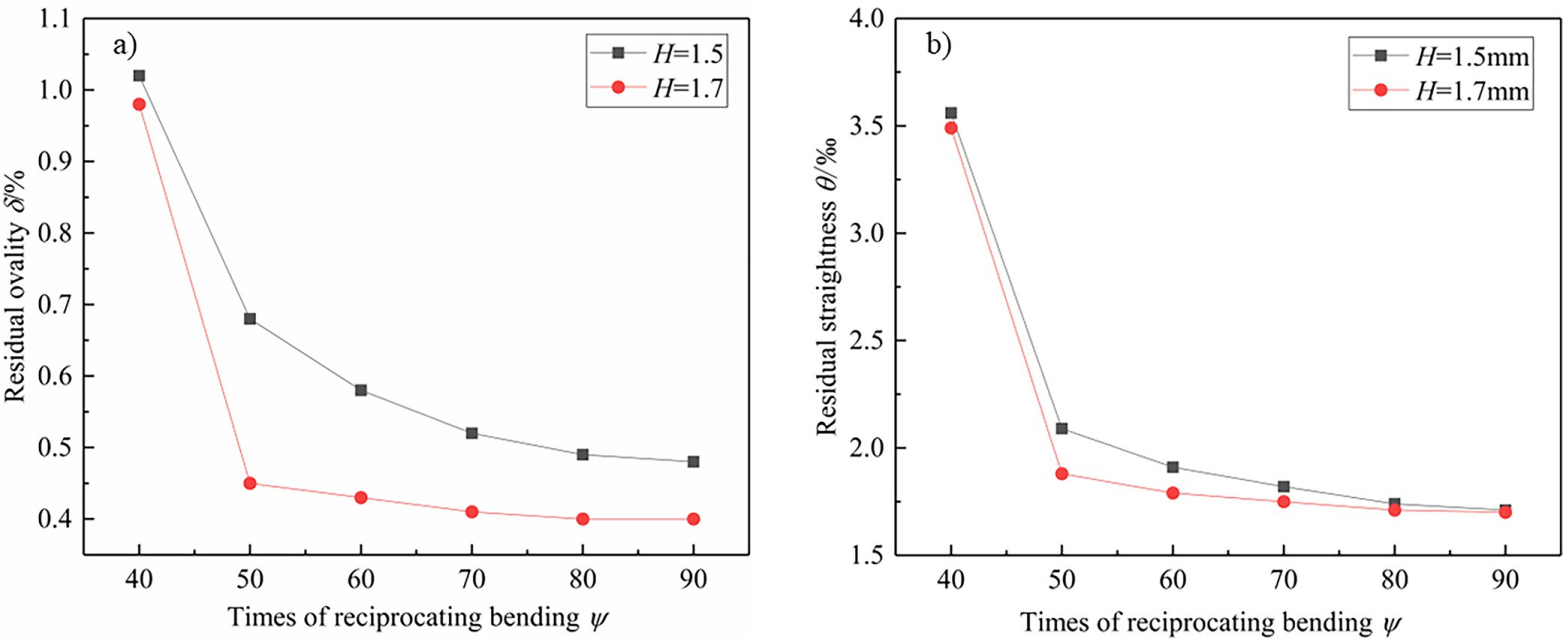
Effect of the times of reciprocating bending on residual ovality and residual straightness of pipes. a) Residual ovality; b) Residual straightness; *t* = 2.0mm.

According to the simulation results, the times of reciprocating bending *ψ* on the residual ovality and residual straightness of pipes is discussed when the *H* is 1.5 mm and 1.7 mm. The ratio of *V*_r_ to *V*_f_ is 20, 25, 30, 35, 40, 45, the number of reciprocating bends is 40, 50, 60, 70, 80, and 90, respectively. The effect of the times of reciprocating bending on residual ovality and residual straightness of pipes is shown in Fig 17.

The residual ovality and residual straightness of pipes gradually decrease with the increase of the times of reciprocating bending. When the times of reciprocating bending is not less than 50, the residual ovality of pipes is within 1% and the residual straightness is within 0.2%. The above results lay the foundation for the development of subsequent calibration schemes.

**Fig 18:**
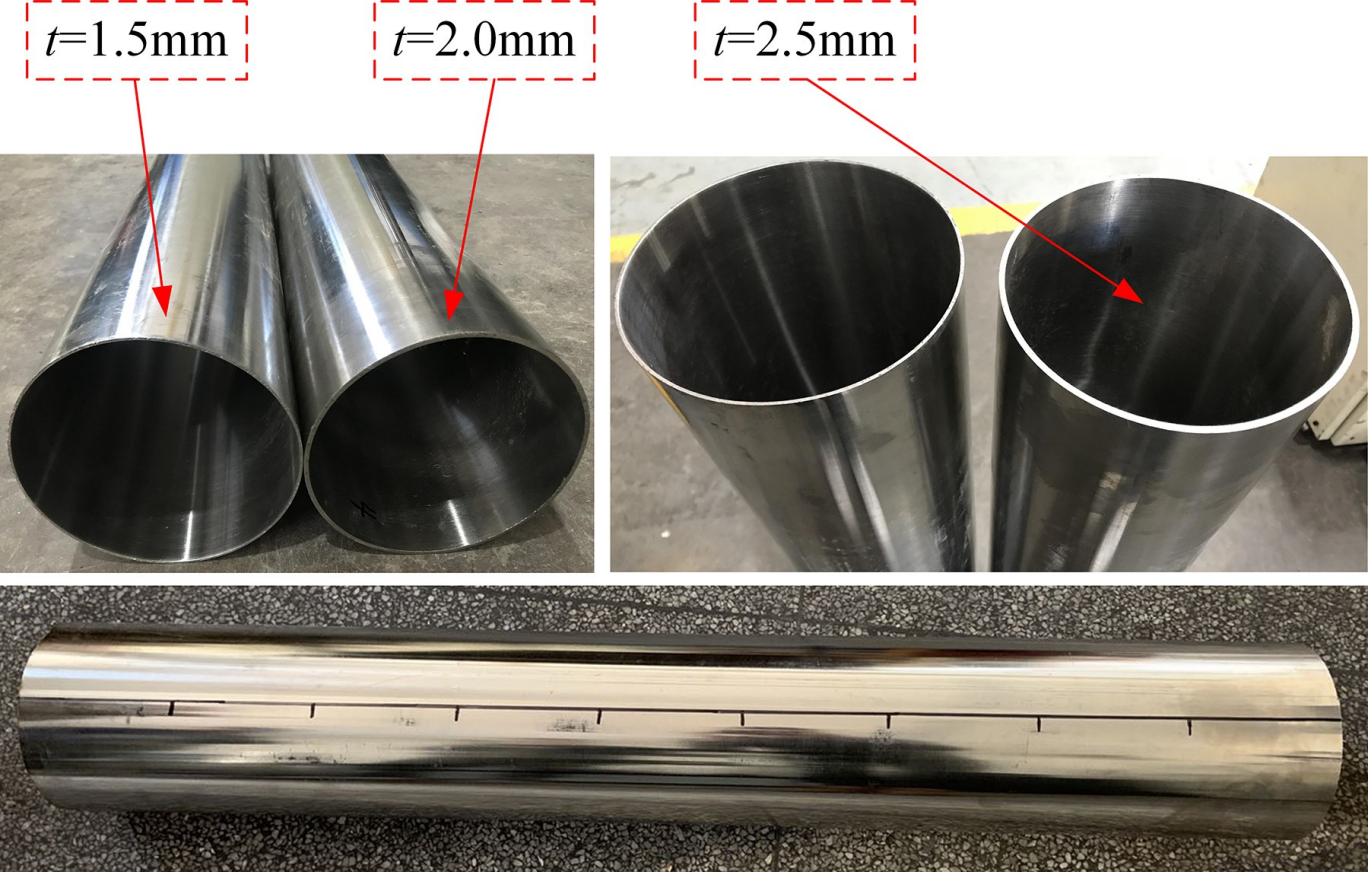
Forming effect of 304 stainless steel pipes.

After calibration, the residual ovality and residual straightness can meet the industry standards [4]. The forming effect of 304 stainless steel pipes is shown in Fig 18.

### Calibration scheme

As discussed earlier, a calibration scheme is proposed by considering process parameters, experimental device, roller-shape, material, and geometric dimension of pipes. It should be noted that the radial reduction, roller rotation speed and pipe forward speed are the direct input parameters of the experiment. It is therefore necessary to determine the above. The calibration scheme is as follows:

1. Loading parameter (radial reduction *H*): For pipes with a diameter of 160 mm with a thickness of 1.5 mm, 2.0 mm and 2.5mm, calibration is available when the radial reduction is 1.7 mm.
2. The ratio of roller rotation speed (*V*_r_) to pipe forward speed (*V*_f_): The ratio of *V*_r_ to *V*_f_ is related to the times of reciprocating bending and the geometric dimension (length and outer diameter) of pipes. Experimental results show that the residual ovality and residual straightness of pipes can meet the industry standard when the times of reciprocating bending is not less than 50. So the ratio of *V*_r_ to *V*_f_ can be calculated in the case of known pipe’s size and the times of reciprocating bending. According to the ratio, *V*_r_ and *V*_f_ can be reasonably assigned within the controlled range of the experimental device operating system.

## Conclusions

1. A new process of continuous and synchronous calibration process of ovality and straightness for LSAW pipes with three rollers is proposed. An experimental device is developed, which can realize the two functions of continuous calibrating ovality and continuous calibrating straightness of LSAW pipes.
2. Through the analysis of shear stress, radial stress, and axial stress, it is concluded that the shear stress is the main stress direction. Three positive bending regions and three reverse bending regions are distributed uniformly along the circumferential direction of the pipe.
3. The influence of process parameters on residual ovality and residual straightness is discussed. That is, the residual ovality and residual straightness of pipes decrease with the increase of the radial reduction and times of reciprocating bending. The reciprocating bending process can eliminate the difference of initial curvature and unify the circumferential and axial curvature to the same direction and value respectively.
4. A calibration scheme based on the process is proposed by using the control variable method. In view of the calibration scheme, the radial reduction, roller rotation speed and pipe forward speed can be determined. It provides guidance for the application of the process in practical production. The residual ovality of pipes is within 1% and the residual straightness is within 0.2%, which complies with the requirements of API standard.

## Supporting information

S1 File (DOCX)
