# Supplementary material for: Continuous and synchronous calibration process of ovality and straightness for longitudinally submerged arc welding pipes with three rollers
Source: PLoS One. 2024 Aug 6;19(8):e0307293. doi: 10.1371/journal.pone.0307293 (PMC11302920; doi:10.1371/journal.pone.0307293)
Supplement: S1 File — (DOCX) [file pone.0307293.s001.docx]

**Fig 1. Schematic diagram of the calibration process**

None

**Fig 2. Schematic diagram of roller-shape**

Diagram of curvature distribution in hyperbolic form：


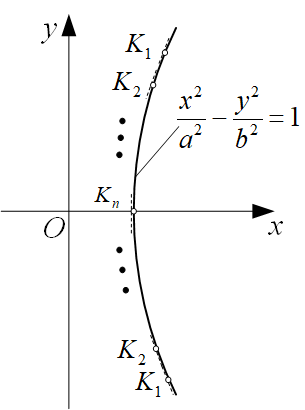


Where, $K=\left|\frac{y''}{\left(1+y'^{2}\right)^{3/2}}\right|$, $\frac{1}{K_{n}}\left|_{x=a,y=0}\right.=\frac{b^{2}}{a}$

**Fig 3. Diagram of loading parameters**

None

**Fig 4. Deformation path of particle along axial direction**

None

**Fig 5. Deformation path of particle along circumferential direction**

None

**Fig 6. Flow chart for formulating the calibration scheme**

None

**Fig 7. Finite element model**

None

**Fig 8. Experimental device for pipe calibration**

None

**Fig 9. Distribution of equivalent stress along the one-third pipe**

None

**Fig 10. Distribution of radial stress along the thickness direction**

None

**Fig 11. Distribution of axial stress along the thickness direction**

None

**Fig 12. Distribution of shear stress along the thickness direction**

None

**Fig 13. Distribution of equivalent strain in the ovality calibration section**

None

**Fig 14. Distribution of residual stress of 304 pipe after calibration**

None

**Fig 15. Effect of the ratio of $V_{\text{r}}$ to $V_{\text{f}}$ on residual ovality and residual straightness**

1. **Description of the experiment**：

To determine the times of reciprocating bending, it is necessary to solve the ratio of roller rotation speed ($V_{\text{r}}$) to pipe forward speed ($V_{\text{f}}$). The ratio is discussed based on numerical simulation analyses.

1. **Methods**：

Control variable method.

1. **Variables**：

The ratio of roller rotation speed ($V_{\text{r}}$) to pipe forward speed ($V_{\text{f}}$).

1. **Experimental results**:

Variable 1: the ratio of roller rotation speed ($V_{\text{r}}$) to pipe forward speed ($V_{\text{f}}$); Result: Residual ovality and residual straightness.

Data1:

| $V_{\text{r}}$(mm/s) | $V_{\text{f}}$(mm/s) | $V_{\text{r}}$/$V_{\text{f}}$ | Residual ovality (%) | Residual straightness (%) |
| --- | --- | --- | --- | --- |
| 188 | 10 | 18.8 | 0.37 | 0.31 |
| 210 |  | 21 | 0.28 | 0.26 |
| 250 |  | 25 | 0.15 | 0.17 |
| 300 |  | 30 | 0.14 | 0.15 |
| 375 |  | 37.5 | 0.13 | 0.13 |

Graph1:



**Fig 16. Effect of radial reduction on residual ovality and residual straightness of pipes**

1. **Description of the experiment**：

The times of reciprocating bending is obtained. Based on the control variable method, the influence of process parameters on the residual ovality and residual straightness is discussed through experiments.

1. **Methods**：

Control variable method.

1. **Variables**：

Radial reduction (*H*); Pipe thickness (*t*).

1. **Experimental results**:

Variable 2: Radial reduction (*H*); Result: Residual ovality and residual straightness.

Data2:

Residual ovality (%):

| Radial reduction (*H*) | Residual ovality (%) | | |
| --- | --- | --- | --- |
|  | *t*=1.5mm | *t*=2.0mm | *t*=2.5mm |
| 0.5 | 1.19 | 1.25 | 1.45 |
| 1 | 1.02 | 1.05 | 1.16 |
| 1.5 | 0.60 | 0.70 | 0.81 |
| 1.7 | 0.45 | 0.46 | 0.50 |
| 2 | 0.41 | 0.45 | 0.48 |
| 2.5 | 0.41 | 0.45 | 0.47 |

Graph2:



Residual straightness (%):

| Radial reduction (*H*) | Residual straightness (%) | | |
| --- | --- | --- | --- |
|  | *t*=1.5mm | *t*=2.0mm | *t*=2.5mm |
| 0.5 | 3.89 | 3.90 | 4.02 |
| 1 | 2.40 | 2.50 | 2.70 |
| 1.5 | 2.02 | 2.13 | 2.45 |
| 1.7 | 1.70 | 1.89 | 1.96 |
| 2 | 1.65 | 1.665 | 1.82 |
| 2.5 | 1.64 | 1.65 | 1.70 |

Graph2:



**Fig 17. Effect of the times of reciprocating bending on residual ovality and residual straightness of pipes**

1. **Description of the experiment**：

The times of reciprocating bending is obtained. Based on the control variable method, the influence of process parameters on the residual ovality and residual straightness is discussed through experiments.

1. **Methods**：

Control variable method.

1. **Variables**：

The times of reciprocating bending ($\psi$).

1. **Experimental results**:

Variable 3: The times of reciprocating bending ($\psi$); Radial reduction (*H*). Result: Residual ovality and residual straightness.

Data3:

Residual ovality (%):

| The times of reciprocating bending ($\psi$) | Residual ovality (%) | |
| --- | --- | --- |
|  | *H*=1.5mm | *H*=1.7mm |
| 40 | 1.02 | 0.98 |
| 50 | 0.68 | 0.45 |
| 60 | 0.58 | 0.43 |
| 70 | 0.52 | 0.41 |
| 80 | 0.49 | 0.40 |
| 90 | 0.48 | 0.40 |

Graph2:



Residual straightness (%):

| The times of reciprocating bending ($\psi$) | Residual straightness (%) | |
| --- | --- | --- |
|  | *H*=1.5mm | *H*=1.7mm |
| 40 | 3.56 | 3.49 |
| 50 | 2.09 | 1.88 |
| 60 | 1.91 | 1.79 |
| 70 | 1.82 | 1.75 |
| 80 | 1.74 | 1.71 |
| 90 | 1.71 | 1.70 |

Graph2:



**Fig 18. Forming effect of 304 stainless steel pipes**

**
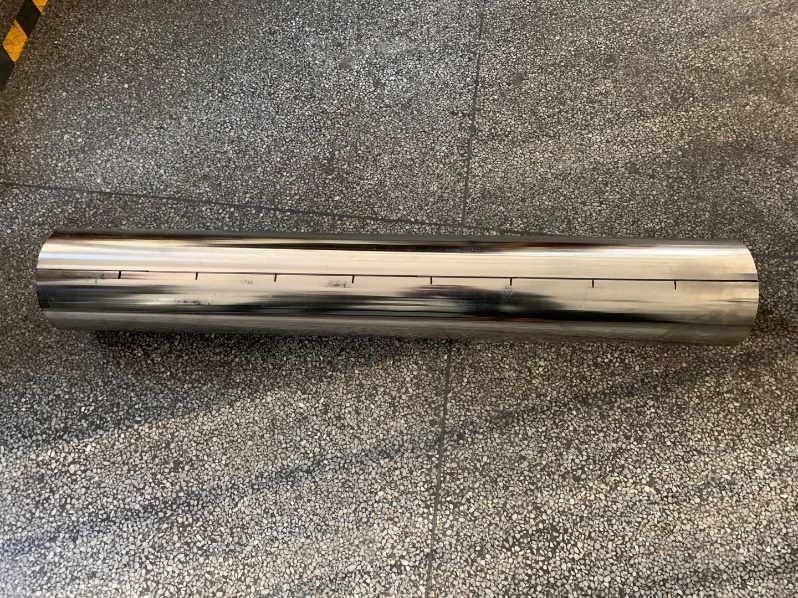

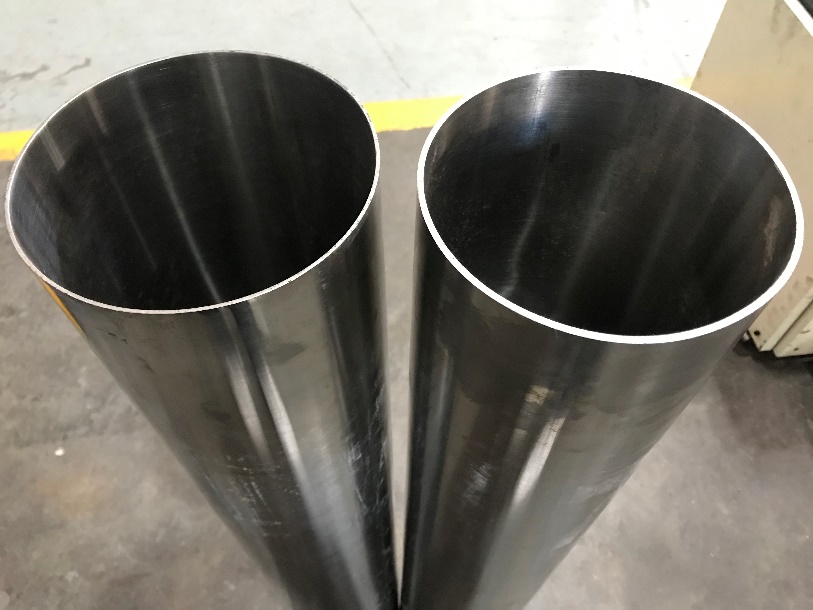

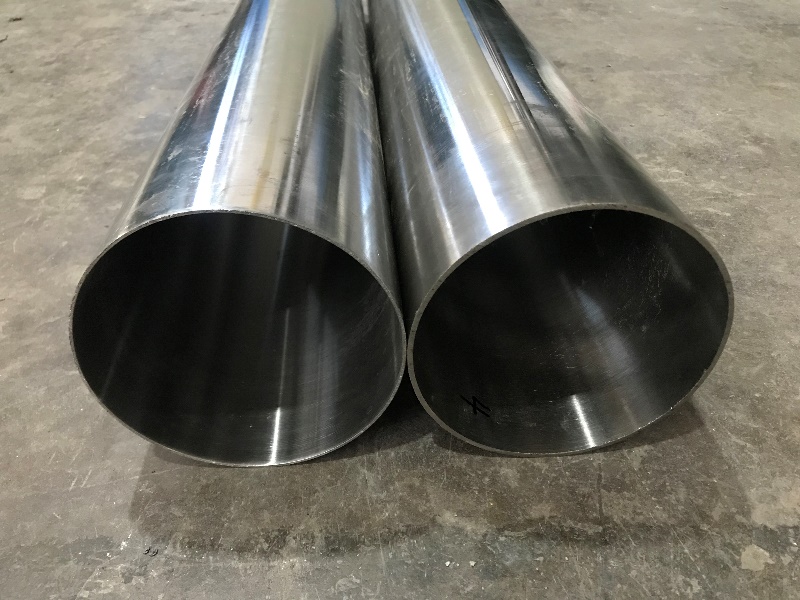
**

**Table 1 Mechanical properties and geometric dimensions of pipes**

| Material | Elastic modulus  *E* (GPa) | Yield stress $\sigma_{\text{s}}$ (MPa) | Plastic modulus  *D* (MPa) | Outer diameter $D_{\text{p}}$ (mm) | Length $L_{\text{p}}$ (mm) | Thickness$t$ (mm) | Initial ovality | Initial straightness |
| --- | --- | --- | --- | --- | --- | --- | --- | --- |
| 304 | 234 | 294 | 2842 | 140/160 | 1000 | 2/1.5 | 5% | 10‰ |

**Table 2 Geometric dimension of rollers**

| Outer diameter $D_{\text{g}}$ (mm) | Length $L_{\text{g}}$ (mm) | Proportion of rollers | Taper of Section Ⅰ (rad) | Taper of Section Ⅴ (rad) | $K_{n}$ (mm^-1^) | Roller shape curve of Section Ⅲ |
| --- | --- | --- | --- | --- | --- | --- |
| 120 | 600 | 1:2:4:2:1 | 0.033 | 0.025 | 0.001 | $0.16x^{2}-0.0004y^{2}-1=0$ |
